# Proton-pumping rhodopsins promote the growth and survival of phytoplankton in a highly variable ocean

**DOI:** 10.1093/ismejo/wrae079

**Published:** 2024-05-02

**Authors:** William G Sunda, Adrian Marchetti

**Affiliations:** Department of Earth, Marine, and Environmental Sciences, University of North Carolina at Chapel Hill, Chapel Hill, NC 27599, United States; Department of Earth, Marine, and Environmental Sciences, University of North Carolina at Chapel Hill, Chapel Hill, NC 27599, United States

**Keywords:** diatoms, rhodopsin, vacuole, photosynthesis, phytoplankton

## Introduction

Proton-pumping rhodopsins (PPRs) were initially found in halophilic archaea [1], and were subsequently observed in marine bacteria [2–4], and in eukaryotic marine phytoplankton, including diatoms, dinoflagellates, haptophytes, and cryptophytes [3, 5, 6]. These membrane proteins contain the embedded pigment retinal, and upon absorption of light, the pigment–protein complex transports a hydrogen ion across the cellular membrane in which the PPR is embedded. The resultant membrane pH gradient can then be used to drive active membrane transport of essential nutrients and biomolecules and to synthesize adenosine triphosphate (ATP), the energy currency of the cell [7, 8].

Recent experiments with the marine diatom *Pseudo-nitzschia subcurvata* isolated from the cold, iron-poor waters of the Southern Ocean (SO) have shown that PPR is localized in the vacuolar membrane of this species [7]. Experiments with this and two other SO diatoms (*Chaetoceros sociales* and *Synedra hyperborea*) showed that cellular PPR levels increased in low-iron cultures [7]. Energetic calculations with the three PPRs containing SO diatoms suggest that light-driven cellular energy production by PPR could substantially augment or exceed that from photosynthesis under certain environmental conditions, namely, high light intensity, cold temperatures, and low availability of the micronutrient iron [7]. At least two of these factors—low temperature and iron limitation—occur in the Southern Ocean [9], where *P. subcurvata*, *C. sociales*, and *S. hyperborea* were isolated and PPR expression is especially high [7].

The vacuole in at least one PPR containing marine algal species represents a second previously unrecognized energy-transducing organelle, which converts absorbed solar energy into utilizable cellular energy. The other organelle is the chloroplast, which through photosynthesis is responsible for the fixation of CO_2_ into organic carbon, which supports virtually all primary production (i.e. life) in the ocean and terrestrial systems [10].

## Proton-pumping rhodopsin photochemistry and photosynthesis are quite different but complementary

**Figure 1:**
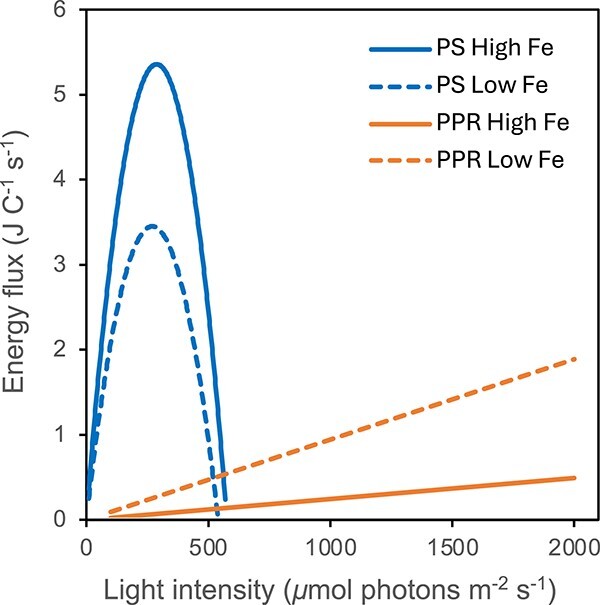
Schematic of the comparative cellular energy production from photosynthesis and PPR photochemistry as a function of light intensity. Photosynthetic energy production rates (joules per Mol cell C per second) were estimated from specific growth rate data for several Southern Ocean phytoplankton isolates grown under increasing light intensities in high-iron (solid lines) and low iron cultures (dashed lines) [7, 12]. The PPR data is taken from estimates of photochemical energy production in the polar diatom *Synedra hyperborea* grown with increasing light intensity in high iron and low iron cultures [7]. Note that while photosynthesis dominates cellular energy production at low light intensities, the opposite should occur at high light intensity due to photo-inhibition of photosynthesis, which is more pronounced in iron-limited cells.

The two light-transducing biological processes—PPR photochemistry and photosynthesis—are, however, quite different in their architecture and functionality, and in many ways, complement one another. Production of cellular energy by PPR involves a simple photochemical process in which the absorption of a photon by its embedded pigment, retinal, leads to the transfer of a proton across the cellular membrane in which the protein is embedded, which for *P. subcuvata*, is the vacuolar membrane. However, photosynthesis is a much more complex process involving three iron-rich membrane-embedded protein complexes: photosystem II (PS II, containing 2–3 Fe), the cytochrome b_6_f complex (5 Fe), and PSI (12 Fe), and the noniron protein nicotinamide adenine diphosphate (NADP) reductase [11]. These protein complexes are connected to one another by motile electron shuttles, two of which (cyt c_6_ and ferredoxin) also contain iron. Each of the two photosystems contain hundreds of light absorbing pigment–protein complexes that function as antennae that funnel absorbed solar excitation energy to a single photoreaction center in PSII and in PSI [11]. Because photoreactions in PSII and PSI are mediated by light absorption by hundreds of antennae pigment–protein complexes, while PPR photochemistry involves light absorption by a single pigment–protein complex, the photosynthetic photosystems (PSII and PSI) absorb much more light than PPR at a given light intensity, and thus, can operate at much lower light intensities. However, this high light absorption makes them much more susceptible to photoinhibition at high light intensities, which is accentuated under iron limitation [11, 12]. In SO phytoplankton grown at 3°C, maximum photosynthetic rates occurred at light intensities of only 60–80 μmol photons m^−2^ s^−1^, only 4%–5% of maximum noon solar intensity (~1500 μmol photons m^−2^ s^−1^ in the SO), and were photo-inhibited at intensities at or above 400 μmol photons m^−2^ s^−1^, and this inhibition was more severe under iron limitation [12] (Fig. 1). PPR, on the other hand, achieves maximum rates at the same temperature at a light intensity of ~2000 μmol photons m^−2^ s^−1^ [7], at or above the maximum solar intensity in the ocean. Consequently, PPR photochemistry should not be photo-inhibited, and PPR:C ratios, and thus PPR photochemical rates, actually increase under low-iron conditions [7]. Calculations show that at low light intensities, photosynthesis should dominate cellular energy production, while at high light, PPR may dominate [7] (Fig. 1) and may provide the cellular energy needed for cellular repair of high-light-induced photodamage to the photosynthetic apparatus.

The existence of two light driven systems for cellular energy production, one (photosynthesis) operating more efficiently at low to intermediate light intensities, while the other (PPR) optimized for high light levels, is beneficial in a world where light intensity varies widely with time of day, season, depth, water clarity, and the extent and variability of cloud cover, with variations occurring on time scales of seconds to seasons. In most regions of the ocean, sunlight undergoes a diel day–night cycle, reaching maximum intensities at solar noon near the sea surface on clear cloudless days. In such a scenario, photosynthesis may dominate cellular energy production near the ocean’s surface in the early morning and evening, while PPR photochemistry may dominate near mid-day when photosynthesis is photo-inhibited. In addition, most oceanic regions have wind-driven surface mixed layers, which can extend below the euphotic zone in turbulent systems, as often occur in the Southern Ocean [13] and in lower-latitude regions during storms. Here, algal cells may be continuously mixed from aphotic depths where neither photochemical process can occur, to shallower depths where photosynthesis dominates, and then to near-surface waters where PPR photochemistry dominates cellular energy production [7]. In addition, in many ocean waters, PPR may dominate cellular energy production at or near the sea’s surface near midday on sunny days, while photosynthesis dominates on cloudy days.

**Figure 2:**
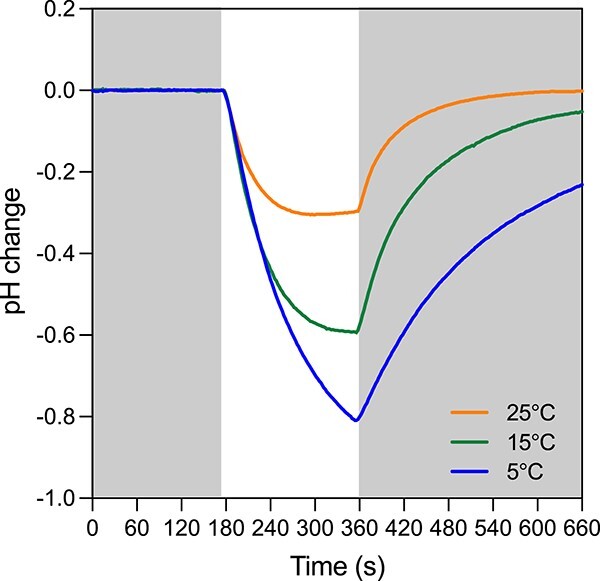
Effect of temperature on the rate of light-driven proton pumping by diatom PPR obtained from the diatom *Pseudo-nitzschia granii*, which was cloned into the plasma membrane of *E. coli*. Outward pumping of protons by PPR was followed using pH measurements in the culture medium with the introduction of light (nonshaded region in the figure), followed by a dark period, where protons leaked from the medium back into the cells across the plasma membrane. Figure reproduced from a previous publication [7].

Temperatures in near-surface ocean waters vary from a low of −1.7°C in cold polar waters to ~30°C in tropical surface waters, which can have a profound effect on the relative contribution of photosynthesis and PPR to cellular energy production. This occurs because of large differences between the molecular architectures of the photosynthetic apparatus and PPR, which lead to major differences in their response to temperature. Because photosynthesis is dependent on motile electron carriers that shuttle electrons between its various protein complex components (PSII, Cyt b_6_f complex, PSI, and NADP reductase), its rate decreases with decreasing temperature, largely due to decreasing diffusion rates of these motile electron carriers [11, 14]. Thus, maximum nutrient-sufficient photosynthetic rates in polar waters are restricted by cold temperatures, relative to rates in warmer temperate and tropical waters [12, 15]. PPR photochemistry, on the other hand, has no motile carrier molecules, and its photochemical rate is limited by its rate of light absorption, which is proportional to light intensity. Thus, PPR photoreaction rates should be independent of temperature, as demonstrated in experiments with diatom PPR cloned into the bacterium *Escherichia coli* [7] (Fig. 2). Furthermore, although initial PPR photo-pumping rates in these experiments were independent of temperature (as predicted from theory [11]) longer-term pH differences across the *E. coli* plasma membrane generated by PPR photochemistry were actually greater at lower temperatures due to lower rates of hydrogen ion diffusion back across the plasma membrane (Fig. 2). Thus, while rates of PPR photochemical reactions appear to be independent of temperature, PPR may actually be able to maintain higher pH gradients across biological membranes (and thus greater net cellular energy production) at colder temperatures because of a reduced diffusion of photo-pumped hydrogen ions back across cell membranes.

A significant difference between photosynthesis and PPR photochemistry is their response to iron limitation, which restricts photosynthetic fixation of carbon in at least ~30% of the world oceans [16] and is especially limiting to phytoplankton growth in the cold waters of the Southern Ocean [9]. As noted earlier, photosynthesis is heavily dependent on iron-rich proteins and protein complexes, including PSI (12 Fe), Cyt b_6_f complex (5 Fe), PSII (2–3 Fe), ferredoxin (2 Fe), and plastoquinone terminal oxidase (2 Fe). Thus, it is heavily dependent on cellular iron, leading to iron-light colimitation of photosynthetic rates at combined low light intensities and low iron concentrations [17–20]. PPR, on the other hand, does not contain iron and hence should not be directly affected by iron limitation [21]. Consequently, PPR photochemistry should be favored relative to photosynthesis in low-iron ocean waters, such as those in the Southern Ocean [6, 7]. Evidence for this is seen in a recent survey of phytoplankton off the West Antarctic Peninsula, where retinal:chlorophyll ratios were highest in iron-poor offshore waters, as were PPR gene expression [7]. In experiments with three separate Southern Ocean diatoms, cellular PPR:C ratios increased in low-Fe cultures [7].

Perhaps the major difference between PPR photochemistry and photosynthesis is the differences in the nature of their final high-energy molecular products. Both PPR photochemistry and photosynthesis generate pH differences across cellular membranes that can then be used to produce adenosine triphosphate (ATP), a motile high-energy compound, which is the energy currency of all cells [11, 21]. However, unlike PPR photochemistry, photosynthesis, through oxidation of water to molecular oxygen, also produces reduced nicotinamide adenine dinucleotide phosphate (NADPH), which, in conjunction with ATP, provides the reducing equivalents and chemical energy to reduce CO_2_ to organic carbon (i.e. carbon fixation) and to support other essential metabolic processes [11]. However, linear electron transfer in photosynthesis (from PSII to Cyt b_6_f, to PSI and finally to NADP) does not produce enough ATP relative NADPH to fix carbon and support cellular growth and metabolism, so additional sources of ATP are needed [22]. The ATP produced from PPR photochemistry can supply the extra-needed ATP to support cellular carbon fixation and growth, and it does so without the need for additional iron. Other cellular processes such as respiration, and photosynthetic cyclic electron transport around PSI and the Cyt b_6_f complex, or around PSII utilizing plastoquinone terminal oxidase, can also supply the extra-needed ATP, but these processes require additional cellular iron [22] and are thus disadvantageous in iron-limited phytoplankton. Thus, in iron-limited marine systems, PPR photochemistry can supplement photosynthesis in supplying the necessary ATP for carbon fixation and cellular growth, without the need for additional iron. The two systems should complement one another in supplying that needed ATP.

## Why is proton-pumping rhodopsin located in the vacuolar membrane?

**Figure 3:**
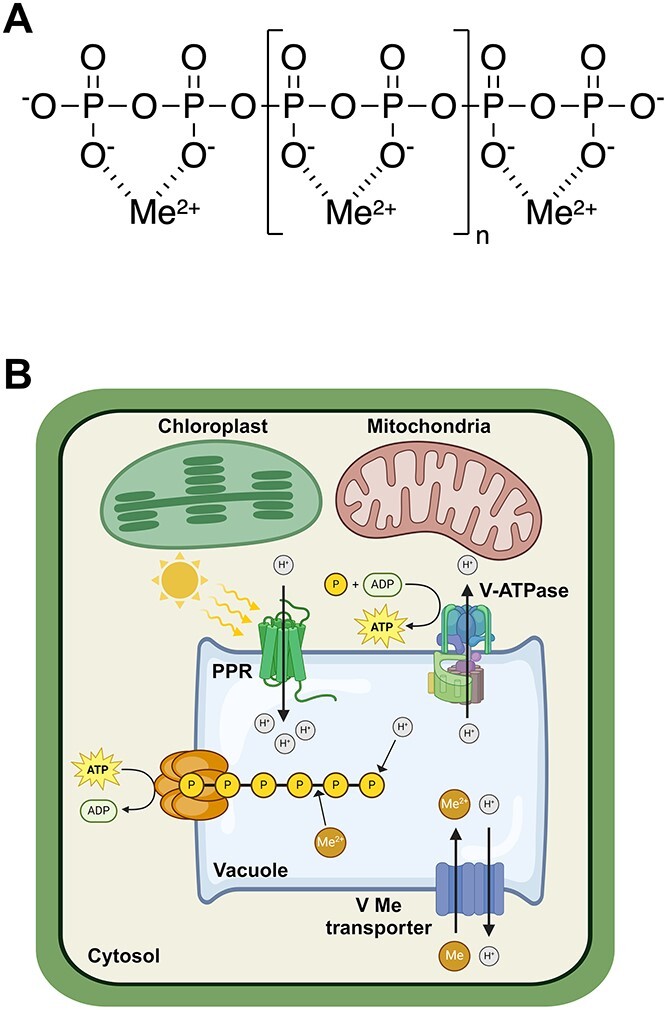
(A) Schematic of a hypothetical polyphosphate chain containing multiple (2n + 4) phosphate groups linked together by high energy phosphoanhydride bonds. The polyphosphate chain contains divalent metal ions (Me) chelated to oxygen atoms on pairs of adjacent phosphate groups. Polyphosphate chains can contain up to hundreds of phosphate groups and can be used to store chemical energy, phosphate, and essential nutrient metals such as iron, manganese, and zinc. (B) Schematic for energy production and storage in the vacuole of a hypothetical PPR-containing marine diatom. The adsorption of light by membrane-bound PPR results in the pumping of protons into the vacuole across the vacuolar membrane. The resulting pH membrane gradient is then used to produce ATP from ADP and inorganic phosphate (P) by ATP synthases or by V-ATPes run in reverse [31]. The resultant cytosolic ATP is then used to produce polyphosphate by a VTC protein complex imbedded in the vacuolar membrane, which simultaneously transports the new polyP chain through the membrane into the vacuolar lumen as it is being formed [32, 33]. In the dark at low levels of ATP, the reaction is reversed and polyP is used to convert ADP back into ATP. Created with BioRender.com.

A question arises regarding why PPR is found in the vacuolar membrane of *Pseudo-nitzschia subcurvata* and likely other marine phytoplankton and PPR-containing eukaryotic microorganisms. To answer that question, we must first inquire about the cellular functions of vacuoles. In many eukaryotic cells, one of the major functions of the vacuole is the storage of excess nutrients not needed for immediate cellular growth requirements [23]. Here, the most notable stored nutrient is phosphate, needed for synthesis of deoxyribonucleic acid, ribonucleic acid, phospholipids, ATP, and other phosphate-containing biomolecules [24, 25]. Excess cellular phosphate in unicellular algae and fungi is usually stored in the vacuole as polyphosphate (polyP) polymers, containing tens to hundreds of phosphate groups linked together by energy-rich phosphate–anhydride bonds [25–27] (Fig. 3A). Traditionally, polyphosphates were thought to mainly serve for the storage of intracellular phosphate accumulated in excess of that needed for immediate cell growth and cell division [24]. But if that were their main cellular function, then cellular polyphosphate concentrations should increase with external phosphate levels and decrease in P-stressed phytoplankton. However, the exact opposite was observed in phytoplankton in the low-P stressed Sargasso Sea, where polyP to particulate (i.e. cellular) P ratios were substantially higher than they were in adjacent ocean waters containing much higher concentrations of dissolved reactive phosphate [28]. Moreover, in experiments in P-limited chemostats, up to 30% of cellular phosphate occurred as polyphosphate in the fresh water alga *Scenedesmus* sp. and that percentage never dropped below 10%, even at the lowest P-limited specific growth rates [29].

Clearly polyphosphate serves other important cellular functions besides storage of excess phosphate. Since the phosphate groups in polyphosphate are linked together by high-energy phosphate anhydride bonds (Fig. 3A) (which are also the high-energy bonds in ATP), a major cellular function for polyphosphates is in energy storage [26, 27]. ATP and polyphosphate undergo the following reaction:


(1) \begin{equation*} \mathrm{ATP}+\mathrm{PolyP_n}\rightleftharpoons \mathrm{PolyP_{n+1}}+\mathrm{ADP} \end{equation*}

as directly shown in bacteria and fungi, where *n* is the number of phosphate residues in the polyP chain [27]. The reaction is reversible, and thus, ATP can be used to produce energy-rich polyP and polyP can be used to convert ADP back into energy-rich ATP. Whether the forward or reverse reaction is favored depends on the relative concentrations of ATP and ADP, and thus, polyP acts as an energy storage system that buffers the intracellular ATP-ADP-Pi balance [27]. Because PPR utilizes sunlight to create a pH gradient across cellular membranes, which then can be used to synthesize ATP [8], the vacuolar membrane is a logical cellular location for evolutionary natural selection to place light-transducing PPR. In that way, the photo-production of a vacuolar membrane pH gradient by PPR can then be used for local production of ATP, which can store cellular energy as vacuolar polyphosphate (Fig. 3B).

In the dark or at low light intensities, when PPR photochemistry is absent or minimal, polyphosphate hydrolysis can supply ATP needed for cellular growth and metabolism. However, at high light intensities where PPR photochemistry is maximal, and photosynthesis is photo-inhibited, the excess cellular energy generated as ATP from PPR photochemistry can then be stored as polyphosphate, to be utilized later at lower light intensities when photosynthesis again dominates cellular energy production, but there is a need for additional ATP for cell growth and metabolism.

In the cell, polyphosphates exist as chelates with divalent metal ions, including those for magnesium, iron, zinc, and manganese [27]. Thus, these polyP chelates can also serve for storage of cellular iron or other essential nutrient metals (e.g. Mn and Zn), when local supplies exceed the immediate cellular needs for growth and metabolism. So, metal polyP chelates serve as effective short-term storage systems not only for phosphate and cellular energy but also for essential metals such as iron, as observed in yeast [30].

Because of its role in short-term nutrient and energy storage, the transport of critical solutes and biomolecules across the vacuolar membrane is highly critical to effective functioning of the vacuolar system and the cell in general. Much of this membrane trafficking involves active transport of phosphate, metal ions, and biomolecules (Fig. 3B). Here, the membrane pH gradient generated by PPR can provide the energy for this essential active membrane transport [31].

Polyphosphate kinase catalyzes the formation and dissociation of polyphosphates [Equation ((1)] in prokaryotes, but in yeast and likely many other unicellular eukaryotes, polyP is produced by a vacuolar transporter chaperone (VTC) protein complex located in the vacuolar membrane. This membrane protein complex synthesizes polyP from ATP on the cytosolic side of the membrane and subsequently transports the new polyP chain through the membrane into the vacuolar lumen [32, 33]. The process requires a low intravacuolar pH, which can be supplied by PPR in the light in PPR-containing cells or by H^+^ V-ATPases in the dark or in cells lacking vacuolar PPR.

Another reason to have PPR photochemistry and photosynthesis segregated into separate organelles is minimizing pigment self-shading (pigment package effects), which is especially problematic in larger cells [11, 23]. Separating photosynthesis and PPR photochemistry in separate cellular organelles lessens shading between retinal in PPR and photosynthetic pigments (chlorophylls and carotenoids). The fact that retinal in many marine phytoplankton (including *P. subcurvata*) adsorbs primarily green light [34], while the main photosynthetic pigment, chlorophyll, has adsorption maxima at red and blue wavelengths, also lessens pigment shading between the two phototrophic pigments.

A vacuolar location for PPR is also seen in the corn smut fungus *Ustilago maydis* [35] and the non-photosynthetic marine dinoflagellate *Oxyrrhis marina* [36]. When the fungal PPR gene was inserted into the yeast *Saccharomyces cerevisiae* (which does not normally contain PPR), the expressed PPR localized to the vacuolar membrane and increased the reproduction rate of the yeast in the light, but not in the dark, indicating that the introduced vacuolar PPR increased cellular fitness [37]. So, in this case, the recombinant PPR in *S. cerevisiae* was localized to the same cellular location as in the native species from which it was derived. However, this behavior does not always occur. In recent experiments, when PPR from the diatom *Pseudo-nitzschia granii* was transferred to the diatom *Phaeodactelum tricornutum* (a non-PPR-containing species), it was localized primarily in the chloroplast, not in the vacuole where it occurred naturally in another *Pseudo-nitzschia* species with an identical PPR gene sequence [34]. Likewise in recent experiments, when the PPR from the SO diatom *Fragilariopsis cylindrus* (whose natural PPR cellular location was not known) was cloned into both *P. tricornutum* and *Thalassiosira pseudonana* (which do not naturally possess PPR), it also localized to the chloroplast [38]. The introduction of PPR into the *T. pseudonana* cells increased cellular growth rate under iron limitation, presumably from PPR photochemical generation of a pH gradient across photosynthetic thylakoid membranes and the resultant production of ATP by ATP synthases [38]. However, as seen in the previous experiments noted above [7, 34], the presence of PPR in the chloroplast of a PPR-cloned species does not necessarily indicate a plastid location in the native species from which the PPR was derived. Clearly, more research is needed to establish the cellular localization of PPR in native PPR-containing phytoplankton to determine if the vacuole or the chloroplast (or both) is the most typical location for the protein.

Although it is highly likely that PPR-generated pH gradients across vacuolar membranes are used for synthesis of ATP in both phytoplankton and fungi, direct evidence for this is currently lacking. Such membrane pH gradients are well known to energetically drive the synthesis of ATP from ADP and inorganic phosphate in chloroplast thylakoid membranes and the inner membranes of mitochondria utilizing the enzyme ATP synthase, which is reversible and can also use ATP to pump hydrogen ions back across the membrane [11]. As noted above, the enzyme H^+^ V-ATPase is used to pump protons into the vacuole in cells not containing PPR or in PPR-containing cells in the dark. Since most V-ATPases are reversible [31], it is likely that at a sufficiently low intravacuolar pH relative to that in the cytoplasm, and high cytoplasmic ADP and Pi concentrations and low cytoplasmic ATP, the V-ATPases will run in reverse and use the pH gradient across the membrane to produce ATP, and thereby promote cellular growth rate [37](Fig. 3B). However, this hypothesis, no matter how likely, currently still needs validation.

## Conclusions

In addition to photosynthesis, PPRs can convert sunlight into utilizable cellular energy as directly shown in experiments that inserted PPR into algal species [38] or yeasts [37] that do not naturally contain PPR. However, the two light-transducing processes are quite different and, in many ways, complement one another. Photosynthesis produces both reducing molecules (NADPH) and high-energy molecules (ATP) needed for carbon fixation, growth, and metabolism, while PPR can only produce membrane pH gradients and ATP, which supplements the ATP produced from photosynthesis or respiration. Furthermore, photosynthetic rates are heavily dependent on the micronutrient iron, decrease with decreasing temperature, operate optimally at low-to-intermediate light intensities, and are photo-inhibited at high light intensities and low iron concentrations while PPR photochemistry does not require iron, is largely independent of temperature, and operates best at high light intensities (Table 1). Thus, given the large range of light intensities, iron concentrations, and temperatures in the ocean’s euphotic zone, the two phototrophic processes should operate optimally under different sets of conditions, with one or the other being favored with changing light levels, which can vary dramatically with time of day, changes in cloud cover, or extent of vertical mixing. PPR was recently found to occur in the vacuolar membrane of a Southern Ocean diatom and in two eukaryotic heterotrophs, making the vacuole a separate light-transducing organelle [35, 39]. Its presence in the vacuole is likely advantageous as cells could use the ATP produced by PPR photochemistry to produce high-energy polyphosphates, an effective energy storage system, although such behavior has yet to be directly demonstrated. We hypothesize that in cold, iron-limited waters at high light intensities, the energy produced by PPR photochemistry could be stored as polyphosphate and drawn on later for needed ATP to support cellular growth and metabolism in the dark or at lower light levels. In this way, PPR photochemistry may be a critical factor supporting the growth and survival of PPR containing marine phytoplankton and other microorganisms, particularly in cold, iron-poor, and deeply mixed marine ecosystems such as those occurring in the Southern Ocean. By benefitting primary production in the Southern Ocean and other iron-poor ocean waters, PPR likely stimulates the biological carbon pump, which transfers atmospheric CO_2_ to the ocean’s interior, helping regulate global climate [16, 40].

**Table 1:** Comparison of characteristics and optimal environmental conditions for energy production between photosynthesis (PS) and proton-pumping rhodopsin (PPR) in marine phytoplankton.

**Variable**	**PS**	**PPR**
Characteristics		
Cellular location	Chloroplast	Vacuole and chloroplast(?)
Primary pigments	Chlorophylls, carotenoids	Retinal
Light-dependent products	ATP and NADPH	Proton gradient and ATP(?)
Environment conditions		
Temperature	Moderate to high	Independent
Light quality	Red and blue wavelengths	Green wavelengths
Light intensity	Low to intermediate	High(?)
Iron demand	High	Independent

(?) indicates further validation needed.

## Data Availability

Data sharing not applicable to this article as no datasets were generated or analyzed during the current study.
